# Role of intravoxel incoherent motion MR imaging in preoperative assessing HER2 status of gastric cancers

**DOI:** 10.18632/oncotarget.17570

**Published:** 2017-05-02

**Authors:** Changfeng Ji, Qinglei Zhang, Wenxian Guan, Tingting Guo, Ling Chen, Song Liu, Jian He, Zhengyang Zhou

**Affiliations:** ^1^ Department of Radiology, Nanjing Drum Tower Hospital Clinical College of Nanjing Medical University, Nanjing, China, 210008; ^2^ Department of Gastrointestinal Surgery, Nanjing Drum Tower Hospital Clinical College of Nanjing Medical University, Nanjing, China, 210008; ^3^ Department of Radiology, Nanjing Drum Tower Hospital Clinical College of Traditional Chinese and Western Medicine, Nanjing University of Chinese Medicine, Nanjing, China, 210008; ^4^ Department of Pathology, Nanjing Drum Tower Hospital Clinical College of Nanjing Medical University, Nanjing, China, 210008

**Keywords:** intravoxel incoherent motion, magnetic resonance imaging, stomach neoplasm, human epidermal growth factor receptor 2, immunohistochemistry

## Abstract

**Purpose:**

To explore the role of intravoxel incoherent motion (IVIM) magnetic resonance (MR) imaging in evaluating human epidermal growth factor receptor 2 (HER2) status of gastric cancers preoperatively.

**Results:**

The apparent diffusion coefficient (ADC) and pure diffusion coefficient (D) values correlated positively with HER2 scores of gastric cancers significantly (*r* = 0.276, *P* = 0.048; *r* = 0.481, *P* < 0.001, respectively). The ADC and D values of HER2 positive gastric cancers were significantly higher than those of HER2 negative tumors (*P* = 0.033, 0.007, respectively). With a cut-off value of 1.321 and 1.123 × 10^−3^ mm^2^/sec, the ADC and D values could distinguish HER2 positive gastric cancers from HER2 negative ones with an area under the curve of 0.733 and 0.762, respectively (*P* = 0.023, 0.011, respectively).

**Materials and methods:**

Fifty-three patients with gastric cancers underwent IVIM MR imaging preoperatively. The values of ADC, D, pseudo diffusion coefficient (D*) and perfusion related fraction (f) of the lesions were obtained. Partial correlation test including tumor volume was performed to analyze correlations between IVIM values and HER2 scores excluding the impact of tumor size. IVIM parameters of gastric cancers with different HER2 status were compared using independent samples *t* test. Diagnostic performance of IVIM parameters in distinguishing HER2 positive gastric cancers from negative ones was tested with receiver operating characteristic analysis.

**Conclusions:**

We confirmed the feasibility of IVIM MR imaging in preoperative assessment of HER2 status of gastric cancers, which might make up the shortfall of biopsy and facilitate personalized treatment for patients with gastric cancers.

## INTRODUCTION

Gastric cancer is one of the most common digestive malignancies worldwide and many patients were diagnosed at advanced stage [1]. Since traditional chemotherapy often encountered resistance with a number of side effects, molecular targeted therapy became another choice for selected patients [2, 3]. Human epidermal growth factor receptor 2 (HER2) is a proto-oncogene encoded by ERBB2 on chromosome 17. When the amplification of HER2 gene occurs, it induces the overexpression of HER2 protein. Then an excessive amount of HER2-containing heterodimers will be formed which enhances the signaling responses to growth factors. With a complex signaling network, the overexpression of HER2 leads to activations associated with cell proliferation, differentiation and survival [4–6]. And HER2 overexpression is an important biomarker for treatment with trastuzumab in patients with gastric and gastroesophageal junction cancers [3]. ToGA trial indicated that the combination of chemotherapy plus trastuzumab proved superior to chemotherapy alone with an extend survival from 11.8 to 16.0 months among patients with higher HER2 expression [3]. Hence, an accurate assessment of HER2 status is critical to optimize the therapeutic effect.

Nowadays, the information of HER2 status is mainly obtained through immunohistochemistry (IHC) or fluorescence *in situ* hybridization (FISH) using biopsy or surgical specimens. However, patients diagnosed at advanced stage usually lose their chance of surgical resection, and endoscopic biopsy can only be used, which is unable to avoid sampling error. In cases with smaller areas of HER2 expression, a biopsy sample taken from a negative area would return a false negative result [7].

During the past few years, magnetic resonance (MR) imaging has been increasingly utilized in preoperative evaluation of gastric cancers [8–10]. Especially diffusion weighted (DW) imaging showed great potential in detection, differentiating diagnosis and features characterization of gastric cancers [11–13]. For instance, the apparent diffusion coefficient (ADC) value correlated significantly with histological differentiation, Lauren classification and TNM staging of gastric cancers [14–16]. Moreover, one previous study reported that the mean and minimal ADC values of HER2 positive gastric cancers were significantly higher than those of HER2 negative ones [17].

However, ADC values derived from traditional DW imaging using 2 b values reflect a combined effect of water molecular diffusion and microvascular perfusion [18]. As an extend model based on DW imaging, intravoxel incoherent motion (IVIM) is able to separate diffusion from perfusion component *in vivo* using an increased number of b values [19]. MR signals obtained at higher b values are mainly related to diffusion, while perfusion effects display dominance at lower b values [20]. With a bi-exponential decay model, the pure diffusion coefficient (D) and pseudo diffusion coefficient (D*) could be obtained simultaneously, along with perfusion related fraction (f). Since its first introduction in 1986 [21], IVIM MR imaging has shown a great potential in characterizing and grading various tumors. For instance, values of IVIM-derived parameters were significantly correlated with histological grade of hepatocellular carcinoma (HCC), and they showed significance in differentiating high-grade from low-grade HCC [22]. Kim Y et al detected significantly lower ADC and D values in HER2 negative breast cancer than positive ones [23]. We hypothesized that IVIM parameters might change based on different HER2 status of gastric cancer, which has never been reported previously.

Therefore, the purpose of this study was to compare the IVIM parameters between HER2 positive and negative gastric cancers, and to explore the role of IVIM MR imaging in evaluating HER2 status of gastric cancers preoperatively.

## RESULTS

**Figure 1:**
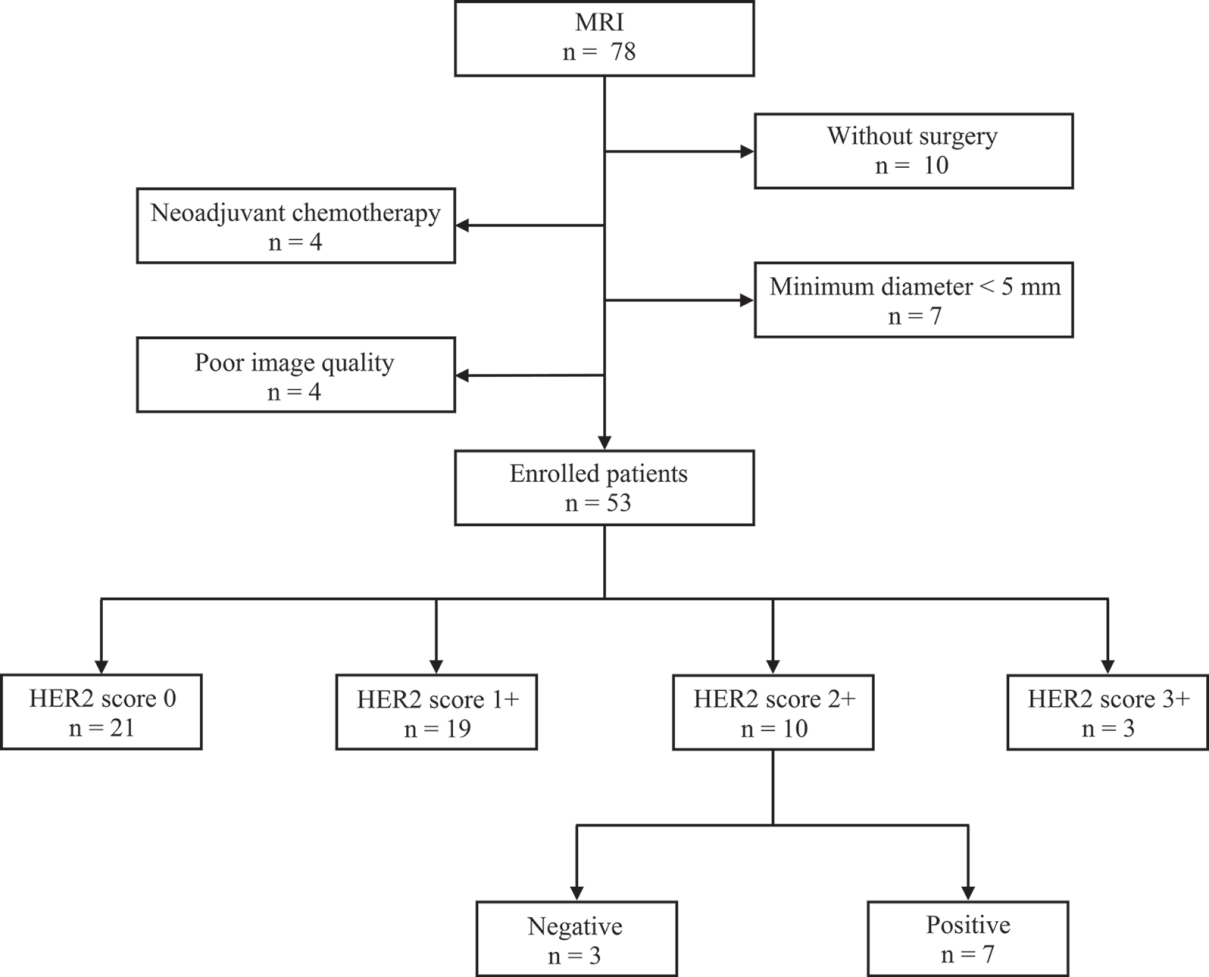
Inclusion and exclusion flowchart of this study

From Nov. 2015 to Oct. 2016, a total of 53 patients with gastric cancers were prospectively enrolled in this study. The patients comprised 32 men and 21 women (age range, 28 ∼ 78 years; mean age ± standard deviation, 60 ± 10 years). A detailed inclusion and exclusion flowchart is shown in Figure 1.

The clinicopathological characteristics of the 53 patients with gastric cancers and different HER2 status are shown in Table 1. Each patient had one lesion identified.

**Table 1:** Clinicopathological information of patients with gastric cancers and different human epidermal growth factor receptor 2 (HER2) status

Characteristics		*n*	HER2 (−) (%)	HER2 (+) (%)	*P* value
Gender	Male	32	25 (78.1)	7 (21.9)	0.722
	Female	21	18 (85.7)	3 (14.3)	
Age	≤ 60 years	22	19 (86.4)	3 (13.6)	0.494
	> 60 years	31	24 (77.4)	7 (22.6)	
Location	Gastroesophageal junction	17	12 (70.6)	5 (29.4)	0.260
	Other stomach	36	31 (86.1)	5 (13.9)	
Pathological type	Ade	38	29 (76.3)	9 (23.7)	0.512
	Pcc	9	8 (88.9)	1 (11.1)	
	Muc	1	1 (100.0)	0 (0.0)	
	Mixed	5	5 (100.0)	0 (0.0)	
Differentiation degree	Poor	34	29 (85.3)	5 (14.7)	0.162
	Moderate-poor	13	11 (84.6)	2 (15.4)	
	Moderate	6	3 (50.0)	3 (50.0)	
Lauren classification	Diffuse type	24	23 (95.8)	1 (4.2)	0.046*
	Mixed type	14	10 (71.4)	4 (28.6)	
	Intestinal type	15	10 (66.7)	5 (33.3)	
T stage	T1	2	1 (50.0)	1 (50.0)	0.565
	T2	7	6 (85.7)	1 (14.3)	
	T3	33	26 (78.8)	7 (21.2)	
	T4	11	10 (90.9)	1 (9.1)	
N stage	N0	6	5 (83.3)	1 (16.7)	0.067
	N1	9	9 (100.0)	0 (0.0)	
	N2	15	9 (60.0)	6 (40.0)	
	N3	23	20 (87.0)	3 (13.0)	
M stage	Mo	50	40 (80.0)	10 (20.0)	0.615
	M1	3	3 (100.0)	0 (0.0)	

Note: Ade, tubular or papillary adenocarcinoma; Pcc, poorly cohesive carcinoma; Muc, mucinous carcinoma; Mixed, adenocarcinoma + poorly cohesive carcinoma (2 cases), poorly cohesive carcinoma + mucinous carcinoma (2 cases), adenocarcinoma + mucinous carcinoma (1 case). *P < 0.05 with chi square test.

**Figure 2:**
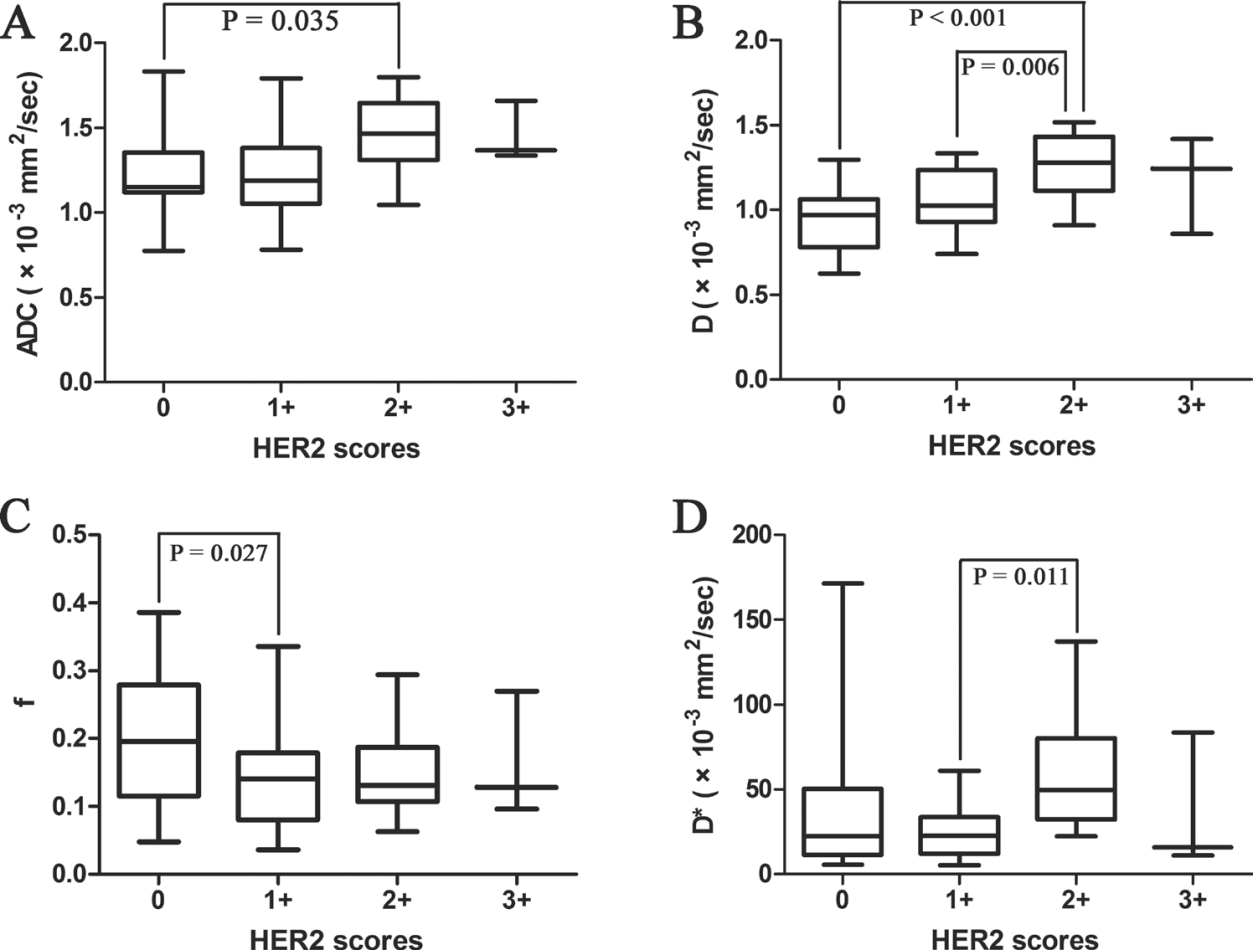
Box plots of the (**A**) apparent diffusion coefficient (ADC), (**B**) pure diffusion coefficient (D), (**C**) perfusion related fraction (f) and (**D**) pseudo diffusion coefficient (D*) values of patients with various HER2 scores (from 0 to 3+). The line within each box represents the median value and the boxes represent data from the 25th to the 75th percentile.

There was significant difference of the D values among gastric cancers with different HER2 scores (*P* = 0.001) (Table 2), especially between score 0 and score 2+ (*P* < 0.001) (Figure 2). And the ADC and D values correlated positively with HER2 scores of gastric cancers significantly (*r* = 0.276, *P* = 0.048; *r* = 0.481, *P* < 0.001, respectively, with partial correlation test including tumor volume).

**Table 2:** Intravoxel incoherent motion magnetic resonance parameters in gastric cancers with different human epidermal growth factor receptor 2 (HER2) scores and status

HER2	ADC	D	f	D*
Score 0 (*n* = 21)	1.245 ± 0.259	0.939 ± 0.186	0.201 ± 0.092	42.404 ± 44.215
Score 1+ (*n* = 19)	1.257 ± 0.251	1.037 ± 0.186	0.141 ± 0.079	24.244 ± 14.465
Score 2+ (*n* = 10)	1.452 ± 0.234	1.254 ± 0.194	0.149 ± 0.066	59.443 ± 34.492
Score 3+ (*n* = 3)	1.455 ± 0.177	1.173 ± 0.285	0.165 ± 0.093	36.822 ± 40.510
ANOVA	*P* = 0.104	*P* = 0.001^†^	*P* = 0.133	*P* = 0.074
Negative (*n* = 43)	1.264 ± 0.254	1.008 ± 0.208	0.168 ± 0.089	34.916 ± 33.486
Positive (*n* = 10)	1.455 ± 0.218	1.212 ± 0.207	0.166 ± 0.073	55.461 ± 40.697
*t* test	*P* = 0.033^‡^	*P* = 0.007^‡^	*P* = 0.959	*P* = 0.099

Note: ADC, apparent diffusion coefficient; D, pure diffusion coefficient; f, perfusion related fraction; D*, pseudo diffusion coefficient. The ADC, D and D* values are in unit of × 10^−3^ mm^2^/sec. ^†^*P* < 0.05 with one-way analysis of variance (ANOVA). ^‡^*P* < 0.05 with independent samples *t* test.

**Figure 3:**
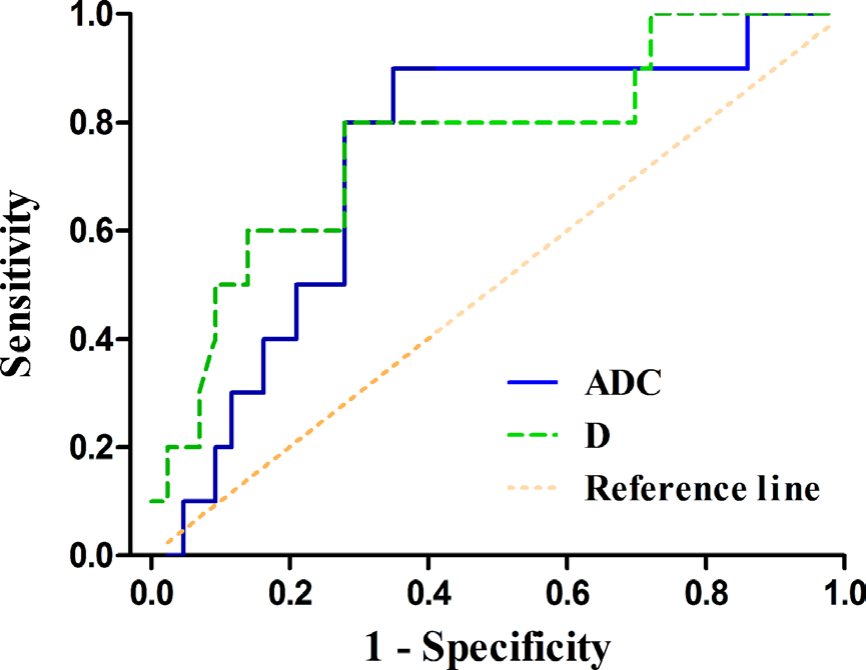
The receiver operating characteristic (ROC) curves of the apparent diffusion coefficient (ADC) and pure diffusion coefficient (D) values in distinguishing HER2 positive gastric cancers from HER2 negative ones (area under the ROC curve, AUC = 0.733, 0.762, respectively) The reference line indicates random assignment.

**Figure 4:**
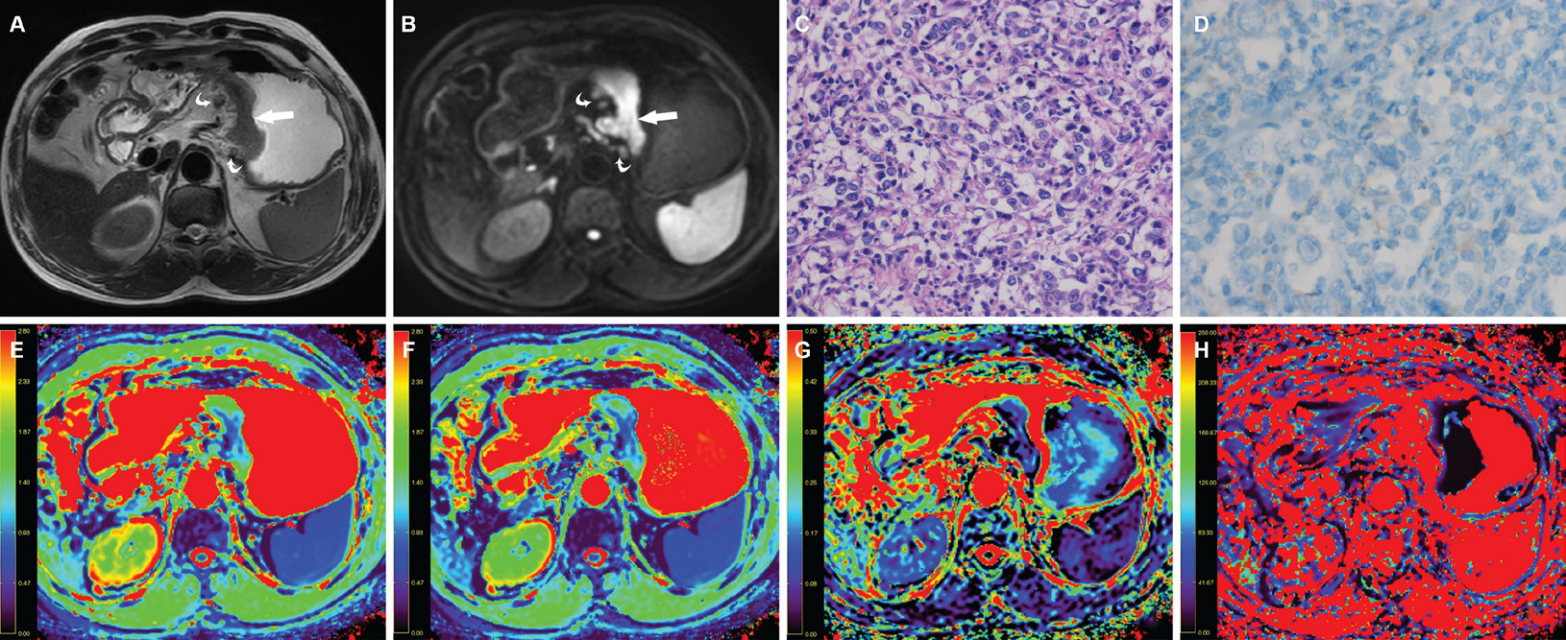
A 63-year-old man with gastric cancer, at stage IIIC (T3N3M0) (**A**) Axial T2 weighted image shows a mildly hyperintense lesion located at the cardia and body of stomach (arrow) and there were enlarged lymph nodes around (curved arrow). The lesion shows hyperintense in (**B**) axial intravoxel incoherent motion (IVIM) magnetic resonance (MR) image (b value = 800 sec/mm^2^) (arrow). (**C**) The photomicrograph of the lesion shows poorly cohesive carcinoma with diffuse type (Hematoxylin & Eosin staining, 200 ×). (**D**) HER2 immunohistochemical assay shows no membrane staining is observed (score 0). The corresponding (**E**) apparent diffusion coefficient (ADC), (**F**) pure diffusion coefficient (D), (**G**) perfusion related fraction (F), and (**H**) pseudo diffusion coefficient (D*) maps show the lesion has an ADC value of 1.131 × 10^−3^ mm^2^/sec, a D value of 0.900 × 10^−3^ mm^2^/sec, a f value of 0.162 and a D* value of 18.876 × 10^−3^ mm^2^/sec, respectively.

**Figure 5:**
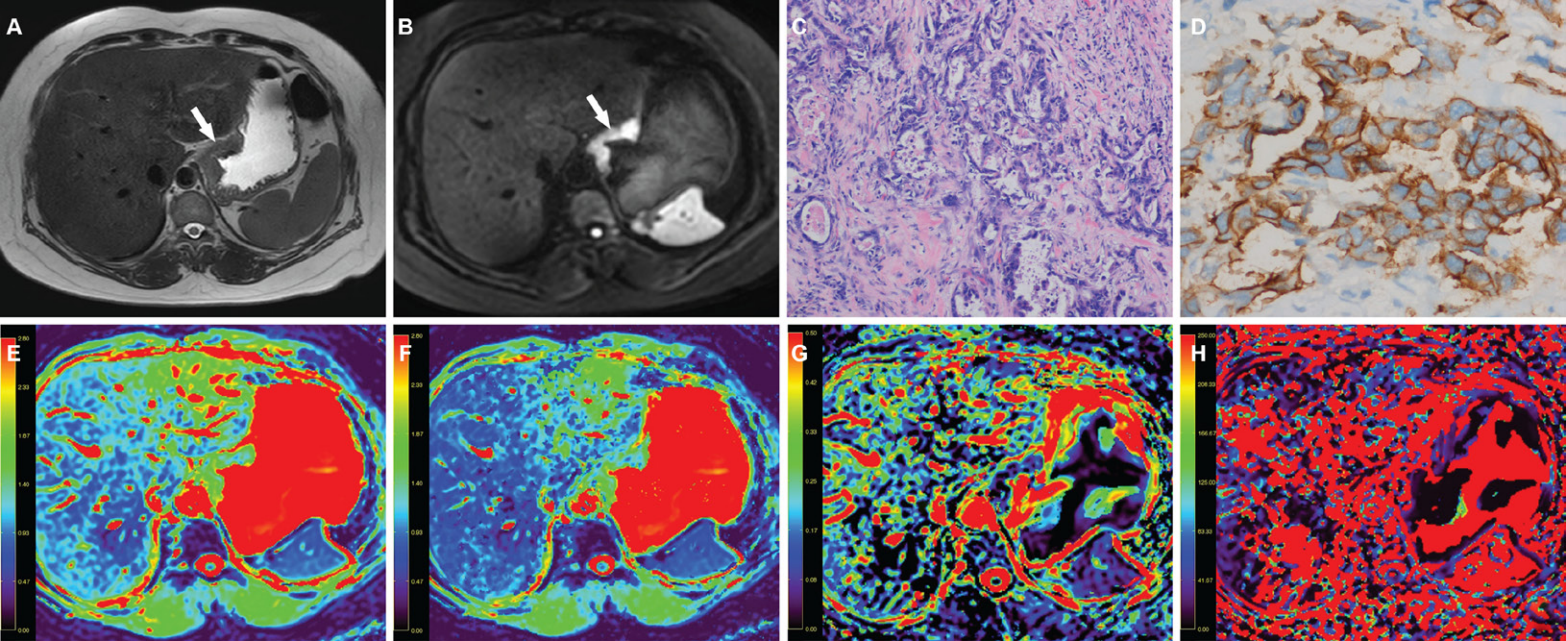
A 51-year-old woman with gastric cancer, at stage IIIB (T3N2M0) (**A**) Axial T2 weighted image shows a mildly hyperintense lesion located at the cardia of stomach (arrow). The lesion shows hyperintense in (**B**) axial intravoxel incoherent motion (IVIM) magnetic resonance (MR) image (b value = 800 sec/mm^2^) (arrow). (**C**) The photomicrograph of the lesion shows poorly differentiated adenocarcinoma with mixed type (Hematoxylin & Eosin staining, 200 ×). (**D**) HER2 immunohistochemical assay shows complete and intense circumferential membrane staining in > 10 % of tumor cells (score 3+). The corresponding (**E**) apparent diffusion coefficient (ADC), (**F**) pure diffusion coefficient (D), (**G**) perfusion related fraction (f), and (**H**) pseudo diffusion coefficient (D*) maps show the lesion has an ADC value of 1.339 × 10^−3^ mm^2^/sec, a D value of 1.242 × 10^−3^ mm^2^/sec, a f value of 0.096 and a D* value of 83.519 × 10^−3^ mm^2^/sec, respectively.

The ADC and D values of HER2 positive gastric cancers were significantly higher than those of HER2 negative tumors (*P* = 0.033, 0.007, respectively) (Table 2). With a cut-off value of 1.321 and 1.123 × 10^−3^ mm^2^/sec, the ADC and D values could distinguish HER2 positive gastric cancers from HER2 negative ones with an area under the curve (AUC) of 0.733 and 0.762, respectively (*P* = 0.023, 0.011, respectively) (Table 3, Figure 3). The representative cases of HER2 positive and negative gastric cancers are shown in Figures 4 and 5, respectively.

**Table 3:** Diagnostic performance of intravoxel incoherent motion magnetic resonance parameters in distinguishing human epidermal growth factor receptor 2 (HER2) positive gastric cancers from HER2 negative ones

Parameters	Cut-off	Sensitivity	Specificity	AUC	*P* value
ADC	1.321	0.900	0.651	0.733	0.023^†^
D	1.123	0.800	0.721	0.762	0.011^†^
f	0.119	0.800	0.395	0.510	0.919
D*	52.352	0.500	0.860	0.670	0.097

Note: ADC, apparent diffusion coefficient; D, pure diffusion coefficient; f, perfusion related fraction; D*, pseudo diffusion coefficient; AUC, area under the receiver operating characteristic (ROC) curve. The cut-off values of ADC, D and D* are in unit of × 10^−3^ mm^2^/sec. ^†^*P* < 0.05 with ROC analysis.

## DISCUSSION

We successfully performed IVIM MR imaging in patients with gastric cancers, and found that preoperative IVIM parameters correlated with HER2 status based on postoperative specimens significantly, which has never been reported previously.

It was reported that HER2 positive gastric cancers were more frequently found in gastroesophageal junction, intestinal type and well differentiated cases [7, 24–28]. In our study, there were no significant differences of HER2 status among different age groups, genders, tumor locations, and differentiation degrees. Although it differed in Lauren classification, our data with only 53 patients was not enough to verify the difference of HER2 positivity in different Lauren classifications. However, a group of studies with large sample size have demonstrated these differences. For instance, Aizawa M et al. reported that higher HER2 positivity was found in well or moderately differentiated gastric cancers than poorly differentiated ones (data from 1,006 patients) [28]. Van Cutsem E et al. reported that overexpression or amplification of HER2 was more common in patients with intestinal histology compared with those with diffuse histology (data from 3,665 patients) [24].

The ADC and D values are parameters reflecting the water molecule diffusion. We found that both ADC and D values correlated positively with HER2 scores of gastric cancers significantly. That is, as HER2 score of gastric cancers increased from 0 to 3+, the ADC and D values also increased gradually. And ADC and D values of HER2 positive gastric cancers were significantly higher than those of HER2 negative tumors, which was consistent with our previous study on traditional ADC values [17]. It was reported that HER2 overexpression tended to be found in well differentiated gastric cancers [28]. As the differentiation degree of gastric cancer decreases, the normal glandular structures are lost. The amount and density of tumor cells increases while their arrangement becomes disordered. A large cell volume and irregular cell shape cause narrower and more distorted intercellular space. As a result, the water molecule diffusion is limited. The loss of cell structure in well differentiated gastric cancers is relatively slight. Thus there are larger spaces for the motion of water molecules in well differentiated tumors, which might lead to higher ADC and D values [14]. A group of studies also reported that HER2 overexpression was more common in intestinal type gastric cancers [24, 29, 30]. Tubular or gland structures are commonly observed in the intestinal type, which may cause relatively large spaces for the motion of water molecules. As a result, intestinal type gastric cancers might show higher ADC and D values.

Moreover, IVIM-derived D value could reflect the motion of water molecules more factually than ADC value for excluding the impact of perfusion effect. Woo S et al. reported that ADC value showed a fair relationship with histologic grade of hepatocellular carcinoma (*r* = −0.448, *P* = 0.002), while *D* value demonstrated a moderate to good relationship (*r* = −0.604, *P* < 0.001) with Spearman correlation test [31]. In our study, the correlation between the D value and HER2 scores (*r* = 0.481) was stronger than the ADC value (*r* = 0.276) with partial correlation test including tumor volume. And our previous study showed that the mean and minimal ADC values correlated with HER2 scores of gastric cancers with *r* values of 0.419 and 0.367 (Spearman correlation test) [17], which were also weaker than the D value in the current study. Both ADC and D values performed well in differentiating HER2 positive and negative gastric cancers with an AUC up to 0.733 and 0.762.

The f value reflects the vascular volume fraction of the tumor, and the D* value reflects the rate of microcapillary blood flow. Both of them represent the perfusion effect, while ADC and D values represent the diffusion effect of tissues [21]. We failed to detect any significant correlation between f or D* value and HER2 scores of gastric cancers in this study. Kim Y et al also reported no significant differences of f and D* values between HER2 positive and HER2 negative breast cancers, while there were significant differences of ADC and D values between them [23]. Ma L et al reported that dynamic contrast enhancement (DCE) MR derived Ve value of gastric cancers with diffuse type was significantly higher than those with intestinal type [32]. However, DCE or perfusion parameters did not necessarily correspond to f value. And the application of D* value was limited due to its instability and low signal to noise ratio [33, 34].

There were some limitations in our study. Firstly, the samples size was relatively small, especially for the cases with HER2 score 3+, which might cause some bias. Secondly, the pathologic foundation and mechanism of higher ADC and D values in HER2 positive gastric cancers were only speculative, without any standard reference. Thirdly, the number and setting of b values were arbitrarily chosen for IVIM imaging without optimization, and the region of interest (ROI) was drawn manually without the reference to postoperative specimen. All the issues would be investigated in our future work.

In conclusion, we confirmed the feasibility of IVIM MR imaging in preoperative assessment of HER2 status of gastric cancers in this study. We established correlations between the ADC as well as D values and HER2 scores of gastric cancers. IVIM MR derived parameters could serve as new biomarkers in predicting HER2 status of gastric cancers, which might make up the shortfall of biopsy and facilitate personalized treatment for patients with gastric cancers.

## MATERIALS AND METHODS

### Patients

Our study received the approval of local ethics committee. Written informed consent was obtained from each patient. The inclusion criteria were: 1) with a diagnosis of gastric cancer confirmed by endoscopic biopsy; 2) willing to undergo MR examination for preoperative assessment; 3) without absolute contraindications to MR examination and gadolinium contrast agents, such as cardiac pacemaker or defibrillator, aneurysm clip, nerve stimulator, insulin pump, cochlear implant. The exclusion criteria were: 1) receiving local or systematic treatment before MR examination or surgery; 2) without accurate HER2 scores and status based on postoperative specimens; 3) with a minimum diameter of tumor less than 5 mm insufficient to contain a ROI; 4) poor MR image quality for further analysis due to motion or magnetic susceptibility artifacts.

### MR examination

All patients underwent MR examination after fasting over 8 hours. After confirming that no contraindications (such as glaucoma, prostate hypertrophy or severe heart disease) were presented, 20 mg of scopolamine butyl bromide (1 mL: 20 mg; Chengdu NO.1 Drug Research Institute Company Limited, Chengdu, China) was injected intramuscularly to prevent gastrointestinal motility 10 minutes before MR examination. Forty-two (79.2%) of 53 patients received scopolamine butyl bromide (no adverse effects occurred within and after MR examination), whereas the remaining 11 patients (20.8%) had a contradiction to the drug regimen (9 patients) or rejected the drug (2 patients). Then all patients were asked to drink 800 ∼ 1000 mL warm water 10 minutes before MR examination to fill the gastric cavity. Before MR scanning, the patients were trained to breathe smoothly.

All MR images were collected by using a clinical whole body 3.0 T scanner (Ingenia 3.0 T; Philips Medical Systems, Best, the Netherlands) with a 32 channels dStream Torso coil. Patients were placed in a supine position with head first. The respiratory sensor was carefully placed between the patient and coil. Scan duration per respiration and respiratory trigger delay were fit into the expiration phase of each patient's respiratory cycle. MR sequences of this study included axial T2 weighted (T2W) imaging, axial IVIM MR imaging and multiphase enhanced T1 high resolution isotropic volume excitation (THRIVE) imaging.

Axial T2W images were obtained with the respiratory-triggered turbo spin-echo sequence without fat-saturation (repetition time, 1000 msec; echo time, 80 msec; matrix, 308 × 252; section thickness, 5 mm; gap, 0.5 mm; field of view, 380 × 380 mm; number of sections, 32; number of signals averaged, 2).

Axial IVIM MR imaging was performed with a respiratory-triggered single-shot spin-echo echo-planar sequence and spectral presaturation inversion recovery (SPIR) techniques (repetition time, 2628 msec; echo time, 55 msec; matrix, 116 × 100; section thickness, 5 mm; gap, 0.5 mm; field of view, 360 × 300 mm; number of sections, 20; number of signals averaged, 3). IVIM MR imaging was acquired with 9 b values (0, 25, 50, 75, 100, 150, 200, 500 and 800 sec/mm^2^) and the sections were enough to cover the lesions.

The THRIVE imaging with breath-holding and spectral attenuated inversion recovery (SPAIR) techniques (repetition time, 3.00 msec; echo time, 1.42 msec; matrix, 224 × 194; section thickness, 5 mm; gap, 0.5 mm; field of view, 380 × 380 mm; number of sections, 32; number of signals averaged, 1) were utilized before and 30, 60, 90 and 180 seconds after administration of 0.2 mL per kilogram of body weight gadodiamide (Omniscan 0.5 mmoL/mL; GE Healthcare, Ireland) using an automatic power injector (Medrad Spectris Solaris EP MR Injector System; One Medrad Drive Indianola, PA, US). All patients underwent MR scanning successfully without any discomfort or side effects.

### Image analyses

All MR images were reviewed by 2 radiologists (J. H., Z. Z.) with 8 and 10 years' experience in abdominal MR imaging, who were blinded to the endoscopic biopsy and postoperative pathologic findings. The IVIM sequence was loaded into a software IDL 6.3 (ITT Visual Information Solutions, Boulder, CO), and then was analyzed with both mono-exponential and bi-exponential models introduced by Le Bihan [19].

Gastric cancer lesions showed mildly hyperintense on T2W, and hyperintense on IVIM (b = 800 sec/mm^2^) images with remarkable contrast enhancement. For each patient, the specific slice of axial IVIM image (b = 800 sec/mm^2^) showing the largest area of tumor was selected. Based on the consensus of two radiologists, an oval ROI (mean area, 49.7 mm^2^; range, 27.2 ∼ 97.8 mm^2^) was manually drawn as large as possible within the solid part of the lesion by referring to the corresponding images of other MR sequences. Artifacts, distortions, vessels, necrotic and hemorrhagic tissues were carefully avoided in choosing the ROIs. If the lesion showed a sandwich sign [12], the ROI was set to avoid the internal muscular layer. Then the ROI was automatically transferred into the parameter maps (ADC, D, f and D* maps, respectively) and the mean value from each ROI was obtained. The ADC value was calculated with a mono-exponential decay model: S_b_ = S_0_ × exp(−b × ADC), by using multiple b values. The D, f and D* values were calculated by the bi-exponential model: S_b_/S_0_ = (1 – f) × exp(−b × D) + f × exp(−b × (D* + D)), in which S_b_ represents the mean signal intensity with diffusion gradient, S_0_ represents the mean signal intensity when b = 0 sec/mm^2^ [19].

### Postoperative pathological analyses

Forty-four patients underwent curative gastrectomy (including 16 total and 28 partial gastrectomies) and 9 patients underwent palliative resection by the surgeons (M. W., H. W.) with 6 and 9 years' experience in gastrointestinal surgery. The pathological analyses were performed by a pathologist (L. C.) with 6 years' experience in digestive malignancy, who was blinded to MR findings and IVIM measurements. The location, histological differentiation and Lauren classification of the gastric cancers were analyzed and recorded according to the World Health Organization (WHO) classification (2010) [35]. Histopathological staging of the tumors was performed based on the TNM classification of the American Joint Committee on Cancer (AJCC, 7th edition) [36]. The mean volume of the tumors was 30617.4 ± 29918.2 mm^3^ (range, 812.5 ∼ 121482.0 mm^3^). In our study, 3 patients (3/53, 5.7%) were diagnosed with distant metastasis (2 with peritoneal metastases and 1 with hepatic metastasis, respectively) postoperatively, which were not observed with conventional diagnosis preoperatively.

A specific scoring system was introduced for the HER2 assessment of the gastric cancers, which was recently reinforced in consensus panel recommendations. The scoring criteria were modified according to the study by Hofmann et al [37]. In detail, when considering HER2 protein status determination using IHC in gastric cancer resections, a patient was classified as score 3+ (IHC positive) if the membrane staining was strong complete, basolateral or lateral in > 10% of tumor cells; score 2+ (IHC equivocal) if the membrane staining was weak-to-moderate complete, basolateral or lateral in > 10% of tumor cells; score 1+ (IHC negative) if the membrane staining was faint/barely perceptible incomplete in >10 % of tumor cells; and score 0 (IHC negative) if no staining was observed or the membrane staining is in < 10% of tumor cells. Equivocal cases at IHC (score 2+) were subjected to FISH analysis. At a cytogenetic level, FISH interpretation criteria were based on a HER2/CEP17 ratio ≥ 2 as a cut off to define a HER2 FISH+ test.

### Statistical analyses

Clinicopathological features of gastric cancers with different HER2 status were compared using chi square test. IVIM parameters of gastric cancers with different HER2 scores were compared using one-way analysis of variance. And the Least-Significant-Difference method was adopted for further multiple comparisons. Partial correlation test including tumor volume was performed to analyze correlations between IVIM values and HER2 scores excluding the impact of tumor size. IVIM parameters of gastric cancers with different HER2 status were compared using independent samples t test. Diagnostic performance of IVIM parameters in distinguishing HER2 positive gastric cancers from negative ones was tested with receiver operating characteristic (ROC) analysis. All statistical analyses were performed with SPSS (version 18.0 for Microsoft Windows ×64, SPSS, US). A two-tailed P value less than 0.05 was considered statistically significant.
